# Comparison of coplanar and noncoplanar intensity-modulated radiation therapy and helical tomotherapy for hepatocellular carcinoma

**DOI:** 10.1186/1748-717X-5-40

**Published:** 2010-05-23

**Authors:** Chen-Hsi Hsieh, Chia-Yuan Liu, Pei-Wei Shueng, Ngot-Swan Chong, Chih-Jen Chen, Ming-Jen Chen, Ching-Chung Lin, Tsang-En Wang, Shee-Chan Lin, Hung-Chi Tai, Hui-Ju Tien, Kuo-Hsin Chen, Li-Ying Wang, Yen-Ping Hsieh, David YC Huang, Yu-Jen Chen

**Affiliations:** 1 Department of Radiation Oncology, Far Eastern Memorial Hospital, Taipei, Taiwan; 2 Department of Surgery, Far Eastern Memorial Hospital, Taipei, Taiwan; 3 Department of Radiation Oncology, Mackay Memorial Hospital, Taipei, Taiwan; 4 Department of Gastrointestinal Division, Mackay Memorial Hospital, Taipei, Taiwan; 5 Department of Medical Research, Mackay Memorial Hospital, Taipei, Taiwan; 6 Institute of Traditional Medicine, School of Medicine, National Yang-Ming University, Taipei, Taiwan; 7 Department of Radiation Oncology, National Defense Medical Center, Taipei, Taiwan; 8 Graduate Institute of Sport Coaching Science, Chinese Culture University, Taipei, Taiwan; 9 School and Graduate Institute of Physical Therapy, College of Medicine, National Taiwan University, Taipei, Taiwan; 10 Department of Healthcare Administration, Asia University, Taichung, Taiwan; 11 Department of Medical Physics, Memorial Sloan-Kettering Cancer Center, New York, NY, USA

## Abstract

**Background:**

To compare the differences in dose-volume data among coplanar intensity modulated radiotherapy (IMRT), noncoplanar IMRT, and helical tomotherapy (HT) among patients with hepatocellular carcinoma (HCC) and portal vein thrombosis (PVT).

**Methods:**

Nine patients with unresectable HCC and PVT underwent step and shoot coplanar IMRT with intent to deliver 46 - 54 Gy to the tumor and portal vein. The volume of liver received 30Gy was set to keep less than 30% of whole normal liver (V30 < 30%). The mean dose to at least one side of kidney was kept below 23 Gy, and 50 Gy as for stomach. The maximum dose was kept below 47 Gy for spinal cord. Several parameters including mean hepatic dose, percent volume of normal liver with radiation dose at X Gy (Vx), uniformity index, conformal index, and doses to organs at risk were evaluated from the dose-volume histogram.

**Results:**

HT provided better uniformity for the planning-target volume dose coverage than both IMRT techniques. The noncoplanar IMRT technique reduces the V10 to normal liver with a statistically significant level as compared to HT. The constraints for the liver in the V30 for coplanar IMRT vs. noncoplanar IMRT vs. HT could be reconsidered as 21% vs. 17% vs. 17%, respectively. When delivering 50 Gy and 60-66 Gy to the tumor bed, the constraints of mean dose to the normal liver could be less than 20 Gy and 25 Gy, respectively.

**Conclusion:**

Noncoplanar IMRT and HT are potential techniques of radiation therapy for HCC patients with PVT. Constraints for the liver in IMRT and HT could be stricter than for 3DCRT.

## Background

Hepatocellular carcinoma (HCC) is one of the most common malignancies worldwide [1] and is the third most common cause of cancer mortality in the recent year [2]. The 5-year survival rate of individuals with liver cancer reported by the American Cancer Society in the United States is less than 10% despite aggressive conventional therapy. In addition, comparing 1991 and 2005, liver cancer is not only one of the three cancers with an increasing death rate, but also the fastest growing death rate (27%) in the United States [3]. Portal vein thrombosis (PVT) is a common complication in patients with advanced-stage HCC, occurring in 20%-80% of these patients [4-6]. PVT may alter the correct evaluation of HCC imaging and also limits HCC treatment choices [7]. The median survival time of HCC patients with PVT is approximately 0.7 to 1.6 months without any treatment [8]. Furthermore, PVT is often a poor prognostic factor for patient survival [9,10].

Several modalities, including surgical resection [11], transcatheter arterial chemoembolization (TACE) [12] and arterial infusion chemotherapy [13], percutaneous ethanol injection therapy, microwave coagulation therapy, radiotherapy, and liver transplantation, have been used in treating HCC [14]. However, there are some limitations to performing these treatments. For example, surgical treatment can only be performed on highly selected patients, because there is a potential risk of postoperative liver failure and early disease recurrence. TACE is considered a contraindication for HCC patients with main portal trunk obstruction and indwelling catheters or catheter-related sepsis, which hinder arterial infusion chemotherapy.

While the role of radiotherapy was limited in the past because of poor tolerance of the whole liver to radiation [15], some studies show that higher irradiation doses resulted in a higher survival rates for HCC patients [16]. Kim DY *et al. *reported a dose-response relationship exists between the radiation dose and PVT, where the objective response of PVT was observed in 3 of 15 patients (20%) with BED < 58 Gy_10 _and in 24 of 44 patients (54.6%) with BED ≧ 58 Gy_10 _[17]. Toya R *et al. *[18] pointed out conformal radiotherapy is effective not only for tumor response but also for survival of HCC patients with PVT. We also reported one HCC patient with PVT who received intensity-modulated radiation therapy (IMRT) with sorafenib, resulting in a significant response and improvement [19]. Moreover, radiotherapy could be an effective treatment choice for selected HCC patients with PVT [20].

With advances in radiotherapy modalities, such as three-dimensional conformal radiotherapy (3DCRT) and IMRT, delivering a good radiation dose to the tumor target volume while sparing the critical organs appears achievable [19,21,22]. With the development of conformal assays, radiation therapy could be an effective choice for selected HCC patients with PVT [20]. Rotational IMRT modalities, including helical tomotherapy (HT) [23], VMAT (Volumetric intensity modulated arc therapy) [19] and the others, are new image-guided intensity-modulated radiotherapy. These complex rotational IMRT machines can deliver highly conformal dose distributions and possess the ability to spare critical organs in a greater extent [19,24]. We evaluated various radiation plans for HT and IMRT as they are currently used at our department. Due to IMRT can preserve acceptable target coverage and better spare nonhepatic organs among HCC patients than 3DCRT [25]. Therefore, we selected different IMRT planning strategies rather than 3DCRT to compare to HT in our study.

The purpose of this study was to define the potential impact of HT and to compare the differences in dosimetric indicators among coplanar and noncoplanar IMRT and HT among HCC patients with PVT previously documented to have at least partial responses to recannularization and to have undergone repeated TACE after IMRT.

## Methods

### Patients

A retrospective study was performed for nine patients with unresectable HCC and PVT underwent step and shoot coplanar IMRT to treat the tumor and portal vein between January 2007 and June 2007, eight of them were men. Patients with at least partial response to RT, documented by identifiable recannularization using CT imaging or abdominal ultrasound, and could be subjected to receive repeated TACE after RT were retrospectively enrolled. All patients had stage IIIA HCC (American Joint Committee on Cancer Staging, 6^th ^edition), chronic hepatitis B carriers and underwent TACE before and after IMRT, with an interval of at least 30 days between the two modalities.

### Radiation therapy

#### (a) Planning CT and Volume definitions

All patients were immobilized using Alpha Cradle^® ^(Smithers Medical Products, Inc. North Canton, OH, USA) in supine position with arms elevated above head to provide a fixed position during CT scan and radiation therapy. Two series of axial CT images, with and without contrast enhancement, with 5-mm contagious slice thickness including whole liver and kidneys were acquired for each patient. Targets were delineated on non-contrast images under the aids by contrast ones. Treatment planning was performed by using non-contrast images. All patients were treated using coplanar static IMRT. No respiratory control or abdomen compression was applied during the treatment, and the organ motion was taken into account in planning-target volume (PTV). Gross tumor volume (GTV) was defined as the hepatic tumor volume plus PVT visualized by contrasted CT images. GTV was expanded by 0.5 cm to create clinical target volume (CTV). A non-uniform three dimensional (3D) margin, 0.5 cm radically and 1.5 cm cranial-caudally was applied to CTV for creating PTV. The normal liver volume was defined as the total liver volume minus the GTV.

#### (b) Dose prescription and planning objectives

The prescription dose was 44.8 to 54.0 Gy depended on the ratio of PTV volume and nonirradiated liver volume [26]. When nonirradiated liver volume was < 1/3, 1/3-1/2 or >1/2 of liver volume, the delivered dose could be 40, 44.8-50.4 or 50-66 Gy, respectively. No patient was given radiation to the entire liver. Treatment was delivered once daily with 1.6 - 1.8 Gy, 5 fractions per week by a 6-MV linear accelerator (Varian 2100IX, Varian Medical Systems, Palo Alto, CA, USA).

For planning objectives, the mean hepatic dose and dose to30% volume of liver was kept less than 30 Gy (V30 < 30%) [18,27-29]. Given that HT is a rotational treatment, volumes of low-dose distributed regions for OARs were generally greater [30]. Thus, volume of normal liver received 10 and 20 Gy (V10, V20) were also investigated for a comparison. For OARs, mean dose to stomach, spleen, kidneys and maximum dose to spinal cord were assessed. The maximum doses were specified as maximum dose to 1% volume, denoted as D1% [31]. According to TD5/5 (the tolerance dose leading to 5% complication rates at 5 years), the mean dose to at least one side of kidney was kept below 23 Gy, and 50 Gy as for stomach [27]. The maximum dose was kept below 47 Gy for spinal cord [27].

#### (c) Description of IMRT and helical tomotherapy techniques

All targets and OARs were delineated on Eclipse V7.3.10 planning system (Varian Medical System, Palo Alto, CA, USA) and then transferred to Helical *Hi-Art *Tomotherapy (Tomotherapy, Inc., Madison, Wisconsin, USA) via Digital Imaging and Communications in Medicine (DICOM) protocol. The dose by IMRT was calculated using the Eclipse system. In Eclipse plans, 5-field gantry arrangement for coplanar and noncoplanar static step and shoot IMRT was designed in all cases. Minimum monitor units (MU) for each segment was set to 5 with no more than 40 segments were allowed for each plan. For HT plans, the field width, pitch, and modulation factor [32,33] used for the treatment planning optimization were 2.5 cm, 0.32 and 3.5, respectively. The dose constraints and the penalties were adjusted accordingly during the optimization process. The dose calculation matrix resolution was 3.0 mm for Eclipse system and 4.0 mm for HT. The inverse planning systems performed iterations during optimization process, which were multi-resolution dose calculation for Eclipse-IMRT but algebraic iteration for HT. For final dose calculation, HT employed convolution/superposition algorithms and Eclipse employed Analytical Anisotropic Algorithm.

#### (d) Conformity index (CI) and Uniformity index (UI)

The dose to PTV has been estimated by DVH after normalization. Dose conformity and homogeneity to the PTV and OARs represent the ability to fulfill dose-volume histogram objectives. The conformity index (CI) was originally proposed by Paddick [34] to evaluate the tightness of fit of the planning target volume to the prescription isodose volume in treatment plans as follows,(1)

where V*_PTV _*is the volume of PTV, V*_TV _*is the treated volume enclosed by the prescriptiond isodose surface, and *TV_PV _*is the portion of the PTV within the prescribed isodose volume. The CI approximates unity means lesser dose to normal tissues and higher dose to target volume. The uniformity index (UI) was defined as *D*_5%_/*D*_95%_, where D_5% _and D_95% _were the minimum doses delivered to 5% and 95% of the planning target volume as previously reported [35]. The greater HI indicates the poorer inhomogeneity.

### Statistical methods

Differences in actuarial outcomes between the three groups were calculated using one-way ANOVA with post hoc multiples comparisons. The differences were considered significant at p < 0.05. All analyses were performed using the Statistical Package for the Social Sciences, version 12.0 (SPSS, Chicago, IL, USA).

## Results

### Target Volume Coverage, Conformity and Uniformity Index

**Figure 1:**
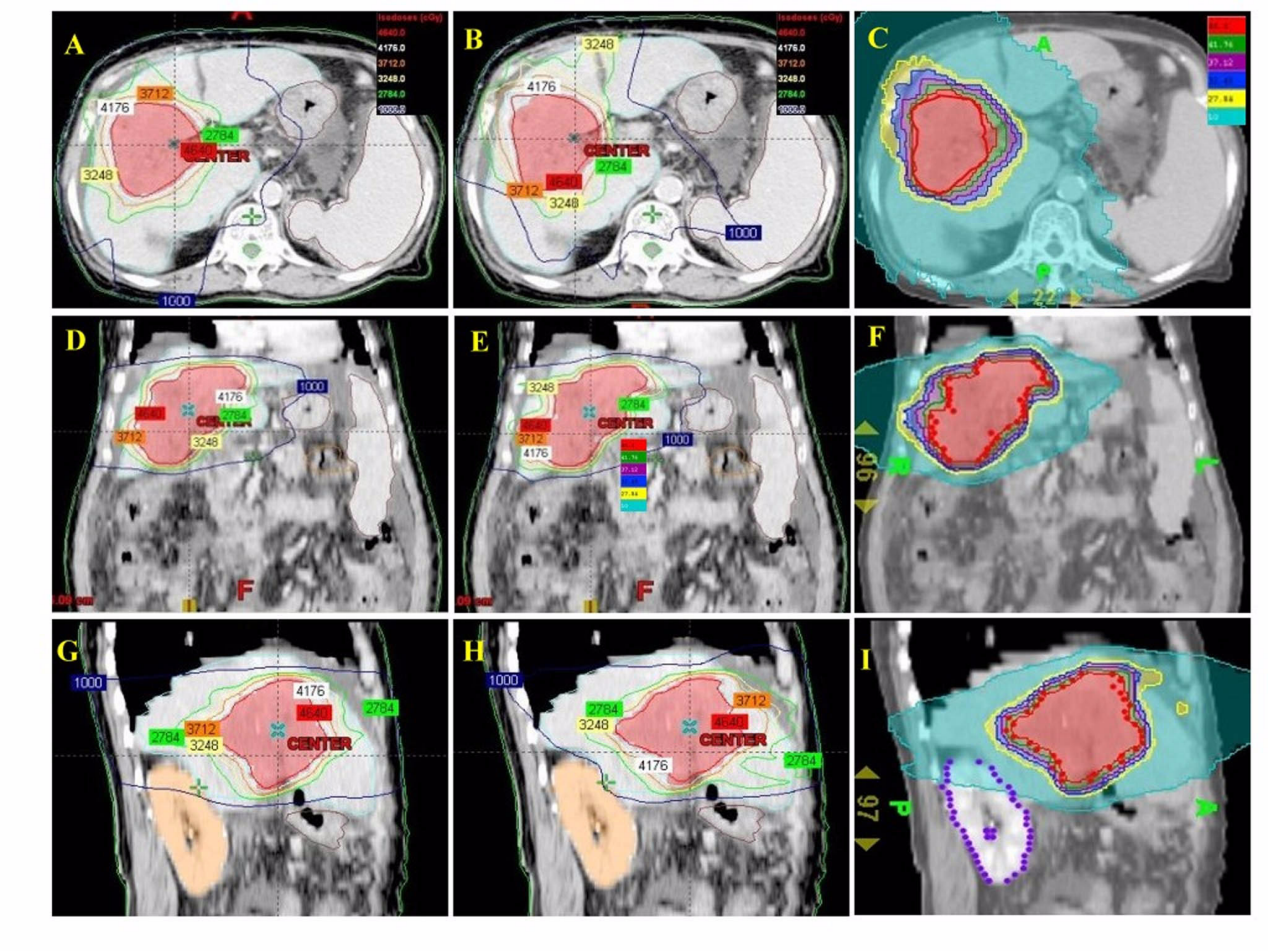
**Isodose distributions of prescribed dose of 46.4 Gy to PTV for different treatment techniques**. A, D and G showed the isodose axial, coronal and sagittal views for coplanar IMRT plan respectively. B, E and H were the isodose axial, coronal and sagittal views for noncoplanar IMRT plan. The helical tomotherapy plan was shown in C, F and I.

**Figure 2:**
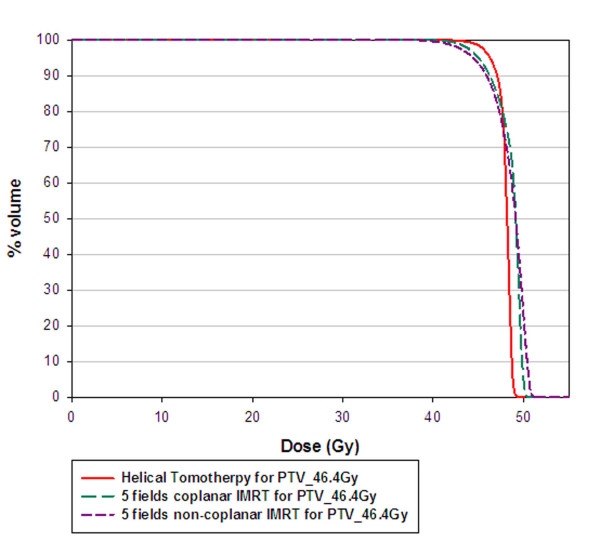
**Dose-volume histogram of planning-target volume for one representative patient undergoing coplanar intensity-modulated radiotherapy (IMRT), noncoplanar IMRT, and helical tomotherapy**.

The average CTV and normal liver volume for the nine patients was 614.4 ± 323.4 ml (range, 154.5-1170.9 ml) and 1294.8 ± 372.9 ml (range, 895.4-2125.8 ml), respectively. The isodose distributions in axial, sagittal and coronal views obtained with coplanar IMRT, noncoplanar IMRT and HT in one representative patient were shown in Fig. 1. Fig. 2 shows dose volume histograms (DVHs) for the PTV of one representative patient using coplanar, noncoplanar IMRT and HT planning techniques. In general, the PTV coverage and comformity was better in HT plan. The similar results were obtained for other patients.

**Figure 3:**
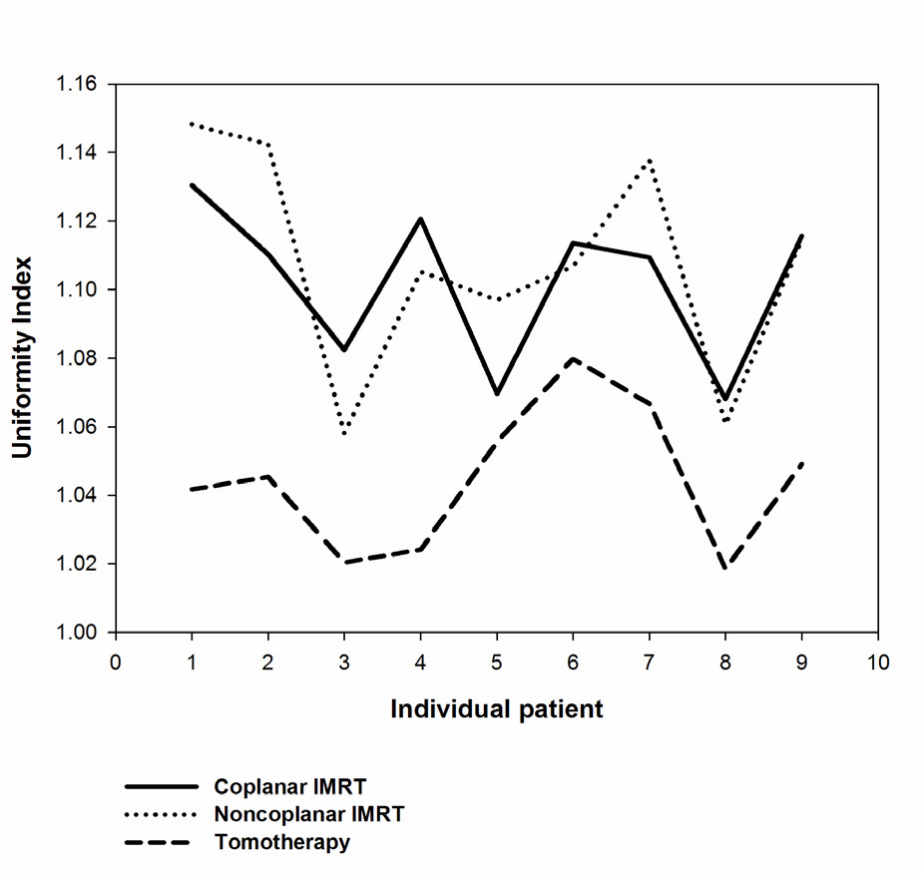
**The uniformity index (UI) for each individual patient undergoing coplanar intensity-modulated radiotherapy (IMRT), noncoplanar IMRT, and helical tomotherapy**.

**Figure 4:**
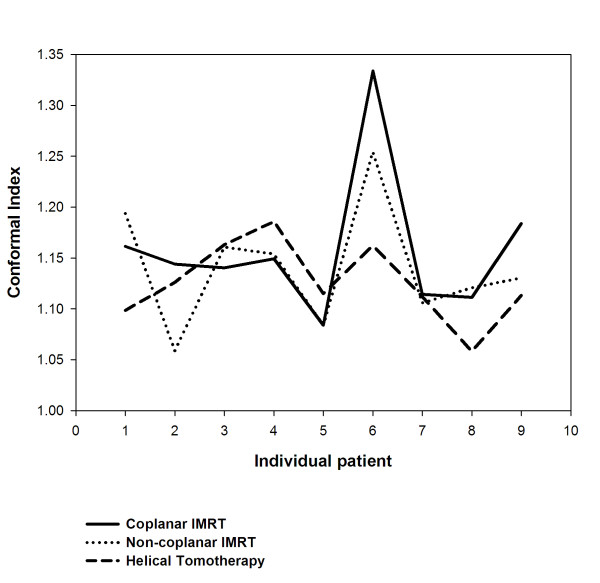
**The conformal index (CI) for each individual patient undergoing coplanar intensity-modulated radiotherapy (IMRT), noncoplanar IMRT, and helical tomotherapy**.

For target coverage, 95% of CTV, 90% and 95% of PTV, all achieved at least 99% of the prescribed dose were listed, respectively. There were no significant differences of coverage for CTV and PTV between three different techniques. (Table 1) The mean score of CI showed no significant difference between the HT and IMRT planning. However, a better uniformity index provided by HT than both IMRT plans was noted (*p *< 0.05) (Table 1). The UI and CI for each individual patient were plotted in Fig. 3 and in Fig. 4, respectively.

**Table 1:** Comparison of dosimetric parameters for irradiation of portal vein thrombosis and target volumes and normal organs at risk (OARs) by using different treatment techniques.

		Coplanar IMRT	Noncoplanar IMRT	Tomotherapy
CTV	V95% (%)	98.72 ± 1.90	99.98 ± 0.02	99.97 ± 0.07

PTV	V90% (%)	99.44 ± 1.07	99.54 ± 0.61	99.84 ± 0.17
	V95% (%)	98.83 ± 0.74	98.71 ± 1.28	99.17 ± 0.64
	UI	1.10 ± 0.02	1.11 ± 0.03	1.04 ± 0.02^#^,*
	CI	1.16 ± 0.07	1.14 ± 0.06	1.13 ± 0.04

Normal liver	V10 (%)	64.81 ± 17.86	51.91 ± 21.56	72.51 ± 13.31*
	V20 (%)	41.36 ± 13.99	32.62 ± 14.95	32.78 ± 9.18
	V30 (%)	21.10 ± 7.90	17.17 ± 8.85	17.00 ± 6.10
	mean (Gy)	18.23 ± 3.11	16.14 ± 4.61	17.93 ± 2.83

Stomach	mean (Gy)	11.68 ± 5.47	9.90 ± 6.18	13.19 ± 6.05

Right Kidney	mean (Gy)	5.07 ± 4.99	6.32 ± 5.08	9.00 ± 8.94

Left Kidney	mean (Gy)	2.1 ± 3.03	2.36 ± 2.91	5.00 ± 5.27

Spinal cord	D1% (Gy)	20.98 ± 7.51	20.12 ± 9.08	22.53 ± 3.31

The Vx is the percentage of normal liver volume that receives ≥ × Gy in the total normal liver volume.

V90 and V95 mean volume covered by 90% and 95% of prescribed dose, respectively.

UI: Uniformity index; CI: Conformal index.

#: The *p *value is < 0.05 for comparing tomotherapy with coplanar IMRT.

*: The *p *value is < 0.05 for comparing tomotherapy with noncoplanar IMRT.

### OARs sparing

The radiation doses for OARs obtained by coplanar IMRT, noncoplanar IMRT and HT were summarized in Table 1. There were no significant differences between both IMRT techniques and HT for the mean doses of liver. The low dose region of liver for HT plans were higher for V10 than others (*p *value < 0.05). There was a trend for noncoplanar IMRT and HT that both techniques provided lower V20 and V30 than coplanar IMRT. For other OARs, there were no significant differences between both IMRT and HT plan for spinal cord, kidneys and stomach (Table 1).

## Discussion

Compared with both IMRT techniques, tomotherapy provides better uniformity. The noncoplanar IMRT technique reduced the normal liver volume receiving 10 Gy to a statistically significant level as compared to tomotherapy. The constraints for V30 of the liver for coplanar IMRT *vs*. noncoplanar IMRT *vs*. tomotherapy might be reconsidered as 21% *vs*. 17% *vs*. 17%, respectively.

Radiotherapy for treating HCC patients has been limited to palliation purpose in the past experiences due to the low tolerance of the whole liver to radiotherapy [36,37] despite HCC being reported as a radiosensitive cancer in clinical investigations [38]. Nevertheless, the radiation dose is the most significant factor associated with tumor response for HCC patients. Troublesomely, as the irradiation doses deliver to the liver increased, hepatic toxicity has become a problem [39]. The encouraging results confirm 3DCRT is an effective modality, not only for tumor response but also for survival in HCC patients who are not suitable for other treatment modalities [17,18,20]. Cheng *et al. *[25] reported that IMRT offers the better potential of increasing the dose conformality to the tumor and reducing the dose to the sensitive structures than 3DCRT does for HCC patients with PVT. Therefore, highly conformality delivered by radiotherapy to HCC patients with PVT cause better tumor control and lower toxicities to normal liver. In the current study, noncoplanar IMRT and HT are compatible with coplanar IMRT in V95 of CTV and PTV. (Table 1) There are no significant differences of CI between HT and both IMRT techniques. However, a trend for more stable conformality for each individual patient provided by HT than both IMRT is noted in Fig. 4. HT provides better uniformity than both IMRT techniques. (Table 1) The UI for each individual patient were plotted in Fig. 3. In addition, the dose-volume histogram for HT had a steeper slope. (Fig. 2) Where the differences among the treatment techniques are clear: suggesting that HT provides higher uniformity within the planning target volume than both IMRT techniques. In summary, HT provides better uniformity for PTV coverage than both IMRT techniques. There is a trend for more stable conformality for each individual patient provided by HT than both IMRT. Nevertheless, both IMRT techniques and HT have similar coverage for V95 of CTV and PTV.

There are several models used to predict liver tolerance, one is the normal tissue complication probability (NTCP) and another one is maximum tolerable dose (MTD). The NTCP model shows that the mean liver dose is the most significant predictor of RILD, with a threshold dose of 31 Gy. The University of Michigan Medical Center reported that the mean hepatic dose was a strong predictor of subsequent radiation-induced liver disease (RILD) and no cases of RILD were observed when the mean liver dose was less than 31 Gy (BED = 30 Gy_10 _in 2 Gy/fraction) [28]. A mean dose to normal liver smaller than 23 Gy (4-6 Gy/fraction) or 30 Gy (2 Gy/fraction) could be safe parameters for RILD prevention as reported by Liang *et al. *[40] and Kim *et al. *[29], respectively (Table 2). The MTD model is based on PTV and liver volume in equal fractions. The concepts of dose constraints for normal organs are extrapolated from the critical volume model [41] as well as the known constraints on partial liver resection that have indicated that up to 80% of the liver can be safely removed in a patient with adequate liver function [42]. In addition, the constraint of 700 cc or 35% of normal liver to receive less than 15 Gy, as no significant instances of RILD have been reported [43].

**Table 2:** The parameters of predicted helical tomotherapy plan within mean 30 Gy to normal liver of hepatocellular carcinoma compared with selected published series.

Published series	Modality	Mean Tumor dose (Gy) /fraction size (Gy)	V30 (%)	Suggested mean dose (Gy) of normal liver under radiotherapy
Dawson *et al. *[28]	3DCRT	52.5/1.5-1.65		30 Gy_10_
Kim *et al. *[29]	3DCRT	54/2		30 Gy
Cheng *et al. *[45]	3DCRT	50/1.8-2	42%	
Liang *et al. *[40]	3DCRT	50/4-6	35%	28 Gy_10_
Yamada *et al. *[44]	3DCRT	57/2	40%	
IMRT	Coplanar IMRT	50.4/1.8	21%	20 Gy
IMRT	Noncoplanar IMRT	50.4/1.8	17%	20 Gy
HT	Tomotherapy	50.4/1.8	17%	20 Gy

*Abbreviations: *3DCRT = Three-dimensional conformal radiotherapy; HT = Helical tomotherapy; V30 = percent volume of normal liver with radiation dose more than 30 Gy; X Gy_10 _= a biologic effective dose (BED) of X Gy_10 _as the α/β ratio = 10 in daily fraction of 2 Gy; OARs = Organs at risk.

Additionally, Yamada *et al. *[44] reported that deterioration of liver function was observed in all patients with V30 > 40%. Chen JC *et al. *suggested that V30 < 42% could avoid RILD [45]. (Table 2) In current study, coplanar IMRT, noncoplanar IMRT and HT provide V30 data as 21%, 17% and 17%, respectively. In the other words, noncoplanar IMRT and HT could be considered as another potential choice for HCC patients with PVT as compared with coplanar IMRT because they can achieve similar dose to the tumor with comparable UI and CI but a relatively low mean dose and V30 to the normal liver. Liang *et al. *[40] reported the tolerable liver volume percentages with 3DCRT planning with hypofractionation (4-6 Gy) was 35% for V25 (= V29 Gy_10_) and 28% for V30 (= V35 Gy_10_). (Table 2) In current study, coplanar IMRT provides similar results for the V10, V20 and V30 of normal liver as 3DCRT planning as compared to the previous report [40]. In the mean time, the noncoplanar IMRT and tomotherapy techniques reduced more than 10% for V20 and V30 of normal liver, respectively. (Table 1) Compared with IMRT, HT has an additional dose superior and inferior to the target volume (Fig. 1) due to the thicker fan beam thickness [46]. Although HT shows greater conformity in the axial view as the dose was delivered rotationally with higher intensity modulation can be achieved. However, HT had greater V10 than the other modalities noted in the current study. (Table 1) The potential risk of radiation toxicities caused by low dose off-targets even with highly conformal radiotherapy has been reported [30]. Careful considerations should be taken into account for the larger low-dose regions to avoid unexpected side effects. According to our results and the guidelines of reducing the potential risk of RILD, we suggest that the constraints for the liver in the V30 for coplanar IMRT *vs*. noncoplanar IMRT *vs*. tomotherapy could be reconsidered as 21% *vs*. 17% *vs*. 17%, respectively. Using IMRT or HT, the constraints for mean dose to the normal liver could be reconsidered as below: when delivering 50 Gy and 60-66 Gy to the tumor bed, the mean dose to the normal liver could be less than 20 Gy and 25 Gy, respectively. The constraints for liver could be more tighten than those used in 3DCRT when we used IMRT or HT for HCC patients with PVT.

The rates of gastrointestinal complications linked to doses of < 40 Gy, 40-50 Gy, > 50 Gy were 4.2%, 9.9%, and 13.2%, respectively [16]. Both IMRT techniques and HT had similar dosimetric effects for OARs. Theoretically, these advantages allow these techniques to push the higher radiation dose to the tumor and keep relatively lower radiation dose to OARs (Table 1).

The applications for reducing liver motion are not used in the current study. To reduce the motion of liver in radiotherapy, several strategies have been reported. The application of four-dimensional computed tomography (4D CT) using an external respiratory signal to acquire difference phases of CT images could improve the dose coverage for target volumes [47]. Further use of abdominal compression was showed effectively in reducing liver tumor motion, yielding small and reproducible excursions in three dimensions [48]. Case *et. al*. [49] showed that the change in liver motion amplitude was minimal over the treatment course and no apparent relationships with the magnitude of liver motion and intrafraction time. The application of 4D CT and abdominal compression may thus increase the coverage of target volume and reduce the motion uncertainty in radiation therapy.

## Conclusions

To sum up, our results suggest that noncoplanar IMRT and HT are potentially effective techniques of radiation therapy for HCC patients with PVT. Constraints for the liver in IMRT and HT could be stricter than for 3DCRT. Further clinical studies of HT and noncoplanar IMRT applied to HCC patients with PVT are warranted.

## Competing interests

We have no personal or financial conflict of interest and have not entered into any agreement that could interfere with our access to the data on the research, or upon our ability to analyze the data independently, to prepare manuscripts, and to publish them.

## Authors' contributions

All authors read and approved the final manuscript. CHH, CYL and PWS carried out all CT evaluations, study design, target delineations and interpretation of the study. CHH drafted the manuscript. CJ C, CCL, TEW, SCL, MJC and KHC took care of patients. HCT, NSC and HJT made the treatment planning and carried out all plans comparisons and evaluations. DYCH and YJ C participated in manuscript preparation and study design. LY W and YPH gave advice on the work and carried out statistical analysis.
